# Natural Deep Eutectic Solvent-Based Dispersive Liquid–Liquid Microextraction Coupled with UHPLC–MS/MS for the Determination of Antibiotic Residues in Food Products

**DOI:** 10.3390/antibiotics15070644

**Published:** 2026-06-27

**Authors:** Ahmed Mostafa, Heba Shaaban, Abdulmalik M. Alqarni, Mansour S. Alturki, Abdulaziz H. Al Khzem, Mohammad A. Alrofaidi, Mohammed Alqarni, Fatimah A. Alansari, Essam M. Hafez

**Affiliations:** 1 Department of Pharmaceutical Chemistry, College of Pharmacy, Imam Abdulrahman Bin Faisal University, King Faisal Road, P.O. Box 1982, Dammam 31441, Saudi Arabia; ammostafa@iau.edu.sa (A.M.); amalqarni@iau.edu.sa (A.M.A.); msalturki@iau.edu.sa (M.S.A.); ahalkhzem@iau.edu.sa (A.H.A.K.); 2 Department of Pharmaceutical Chemistry, Faculty of Pharmacy, Al-Baha University, Al-Baha P.O. Box 1988, Saudi Arabia; malrofaidi@bu.edu.sa; 3 Department of Pharmaceutical Chemistry, College of Pharmacy, Taif University, P.O. Box 11099, Taif 21944, Saudi Arabia; m.aalqarni@tu.edu.sa; 4 College of Pharmacy, Imam Abdulrahman Bin Faisal University, Dammam 31441, Saudi Arabia; 2220001110@iau.edu.sa; 5 Forensic Medicine and Clinical Toxicology Department, Faculty of Medicine, Minia University, Minia 61519, Egypt; essam.mohamed1@mu.edu.eg; 6 Dammam Forensic Toxicology Center- DFTC, Ministry of Health, Dammam 31413, Saudi Arabia

**Keywords:** antibiotics, LC-MS/MS, natural deep eutectic solvents, NADES, dispersive liquid–liquid microextraction, DLLME, sustainable chemistry, green analytical chemistry, GAC, greenness assessment

## Abstract

Background/Objectives: The application of green analytical chemistry (GAC) principles is increasingly important in developing sustainable analytical practices for food safety monitoring. Natural deep eutectic solvents (NADESs) have emerged as green alternatives to conventional organic solvents. This study aimed to develop a sustainable analytical method for determining antibiotic residues in processed meat and frozen poultry products. Methods: A dispersive liquid–liquid microextraction (DLLME) procedure based on a NADES composed of anisaldehyde and decanoic acid (3:1, molar ratio) was coupled with UHPLC–MS/MS for the simultaneous determination of macrolides (clarithromycin, erythromycin), sulfonamides (sulfamethoxazole, sulfadimethoxine), and a fluoroquinolone (enrofloxacin) in food samples. Key extraction parameters, including NADES volume, vortex time, centrifugation time, sample amount, and pH, were optimized. The method was validated for linearity, accuracy, precision, and recovery and applied to real samples from the Saudi market. Results: The method showed excellent analytical performance, with good linearity (R^2^ ≥ 0.9982), recoveries of 84.1–99.4%, and RSDs ≤ 5.75%. The target antibiotics were successfully quantified in processed meat and frozen poultry samples, confirming applicability. In addition, a comprehensive evaluation using eight assessment tools confirmed the method’s environmental sustainability, practicality and innovation. Conclusions: The proposed NADES-based DLLME–UHPLC–MS/MS method is a rapid, sensitive, and eco-friendly alternative to conventional techniques for monitoring antibiotic residues in processed meat and poultry, supporting both food safety and GAC principles.

## 1. Introduction

Antibiotics are vital in both human and veterinary medicine, playing a key role in the treatment and prevention of bacterial infections. Their use has greatly improved health outcomes and saved countless lives, including those of livestock. However, the extensive and sometimes excessive application of antibiotics in veterinary settings has raised concerns about residues in food products derived from animals [1]. A major factor contributing to this issue is the off-label use of antibiotics at sub-therapeutic levels in animal feed, aimed at promoting growth and controlling infections in densely populated livestock environments [2,3]. To mitigate the associated public health and environmental risks, the European Union (EU) has prohibited the use of antibiotics at sub-therapeutic doses as feed additives [4].

Chicken and other meat products rank among the most widely consumed animal-based foods worldwide. However, contamination of these foods with antibiotic residues presents serious public health concerns. Residual antibiotics can exert toxicological effects, trigger allergic or immunological reactions, and disrupt the human microbiome [5]. Furthermore, the presence of sub-therapeutic levels of antibiotics in food contributes to the emergence and proliferation of antibiotic-resistant bacteria, posing a critical challenge to global health and complicating the treatment of infectious diseases [6]. To address these concerns, the European Union (EU) introduced regulations establishing maximum residue limits (MRLs) for antibiotics and other veterinary drugs across a range of food matrices [7]. The successful implementation of these MRLs as a benchmark for evaluating the safety of animal-derived food products has influenced regulatory practices beyond the EU. For example, the Gulf Cooperation Council has established strict regulations for product classification, aiming to improve food quality and safeguard consumer health in the gulf region [8].

Traditional sample preparation techniques have been extensively used to determine antibiotic residues in different food matrices including liquid–liquid extraction and solid-phase extraction [9]. However, both approaches have notable limitations such as high solvent consumption, time-intensive procedures, and in some cases, insufficient selectivity or recovery efficiency [10].

To promote environmental sustainability in pharmaceutical analysis, various green strategies have been implemented, focusing on reducing hazardous solvent use, minimizing waste, and improving overall method efficiency [11,12]. To overcome the limitations of conventional extraction methods, miniaturized extraction techniques, such as dispersive liquid–liquid microextraction (DLLME), are highly advantageous, as they not only reduce solvent consumption but also shorten extraction time, enhance extraction efficiency, and offer simplicity, cost-effectiveness, and environmental sustainability [13]. Despite its advantages, selecting appropriate extracting solvents continues to be a major challenge [13]. Conventional DLLME methods frequently utilize toxic organic solvents, creating potential hazards for both human health and the environment. Various strategies have been developed to render analytical methods greener and more sustainable, ensuring compliance with the principles of green analytical chemistry (GAC) during method development [14,15].

Recently, environmentally friendly deep eutectic solvents (DESs) have attracted considerable interest as a sustainable and effective option for DLLME. Deep eutectic solvents (DESs) are liquids at or close to room temperature, produced by combining hydrogen bond donors (HBDs) and hydrogen bond acceptors (HBAs) in defined molar ratios [16]. The hydrogen bonding between the HBD and HBA components markedly reduces the melting point of the mixture, creating a eutectic system that remains liquid under ambient conditions [17]. Although DLLME significantly reduces the overall consumption of organic solvents, the extractant phase often still contains toxic solvents [18]. Consequently, current research has increasingly focused on developing greener alternatives that can replace conventional toxic solvents, ensuring both high analytical performance and improved environmental sustainability. A particularly promising green alternative to traditional extractants in DLLME is the use of natural deep eutectic solvents (NADESs). These solvents are highly regarded for their adjustable physicochemical characteristics and their distinctive selectivity toward a broad spectrum of analytes. By carefully selecting and varying the types and ratios of the hydrogen bond donors (HBDs) and acceptors (HBAs), NADESs can be tailored to maximize extraction efficiency and achieve optimal selectivity for specific target compounds [16].

NADESs are generally composed of readily available, biodegradable, and low-toxicity components, including organic acids, alcohols, and amines, making them environmentally friendly. These properties render NADESs an appealing and sustainable substitute for conventional organic solvents in DLLME [16]. NADES-DLLME has been extensively applied for the analysis of food [19,20,21], environmental [22,23], and personal care products [24].

The present study aims to develop and optimize a NADES-based DLLME procedure for the extraction and quantification of five antibiotic residues, clarithromycin (CLR), erythromycin (ERY), sulfamethoxazole (SMX), sulfadimethoxine (SDM) and enrofloxacin (ENR), in various food samples including processed meat and frozen poultry products. These compounds were selected to represent major antibiotic classes commonly used in poultry production, including macrolides, sulfonamides, and fluoroquinolones, providing coverage of environmentally and clinically relevant antibiotic groups in the investigated matrices. To demonstrate that the proposed method aligns with recognized greenness standards, a comprehensive greenness evaluation was conducted. Employing multiple assessment tools offers a more complete and reliable picture of the method’s overall environmental friendliness [25]. In this current work, the greenness of the proposed method was evaluated using five established assessment tools, namely the Analytical Eco-Scale (AES) [26], Green Analytical Procedure Index (GAPI) [27], Analytical GREEnness Metric (AGREE) [28], Analytical GREEnness Metric for Sample Preparation (AGREEprep) [29], and the Modified GAPI (MoGAPI) [30]. In addition to evaluating greenness, the applicability and practical performance of the method were assessed using the Blue Assessment of Green Index (BAGI) [31] and the Click Analytical Chemistry Index (CACI) [32]. Furthermore, the level of innovation of the developed approach was evaluated using the recently introduced Innovative Method Orange Gradient Index (IMOGI) [33].

## 2. Results and Discussion

### 2.1. Synthesis of Anisaldehyde-Based NADESs

To identify an effective hydrophobic NADES, seven binary mixtures were prepared by combining anisaldehyde with various hydrogen bond donors including acetic acid, formic acid, oleic acid, octanoic acid, decanoic acid, thymol, and fenchone in a 1:1 molar ratio. Each NADES formulation was then assessed for its extraction capability. Among the tested systems, the anisaldehyde–decanoic acid mixture demonstrated superior performance and was therefore chosen for continued development.

**Figure 1:**
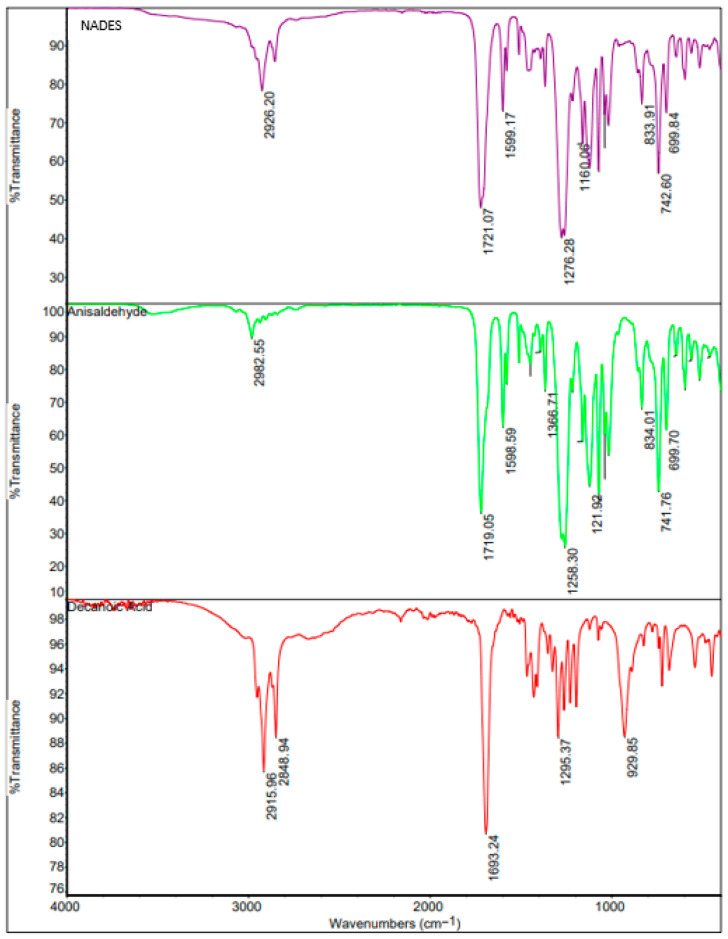
FTIR spectra of anisaldehyde–decanoic acid NADES and its constituents.

FTIR spectroscopy confirmed NADES formation and elucidated the intermolecular hydrogen-bonding interactions by comparing spectra of the pure components with the formed mixture. Pure anisaldehyde exhibited characteristic aromatic C−H stretching at 2982.55 cm^−1^, C=O stretching at 1719.05 cm^−1^, and aromatic C=C bands at 1598.59 and 1366.71 cm^−1^. Pure decanoic acid showed aliphatic CH_2_ asymmetric and symmetric stretching at 2915.96 and 2848.94 cm^−1^, a carboxylic C=O stretch at 1693.24 cm^−1^, and a broad O−H stretching absorption spanning 3300–2500 cm^−1^ characteristic of the hydrogen-bonded carboxylic acid dimer. Upon NADES formation, four diagnostic spectral changes collectively confirmed the establishment of an intermolecular O−H···O=C hydrogen bond between decanoic acid (HBD) and anisaldehyde (HBA: (i) the broad O−H stretching band of decanoic acid underwent significant attenuation, indicating disruption of the self-associated dimer and engagement of the −OH group in a new intermolecular hydrogen bond; (ii) the C=O stretching band shifted from 1693.24 cm^−1^ (pure decanoic acid) to 1721.07 cm^−1^ in the NADES (+27.83 cm^−1^), reflecting redistribution of electron density within the carbonyl system upon proton donation; (iii) the aldehyde C=O of anisaldehyde shifted slightly from 1719.05 to 1721.07 cm^−1^, consistent with perturbation of the carbonyl environment upon acting as HBA; and (iv) the C−O stretching region showed new absorptions at 1276.28 and 1160.46 cm^−1^, differing from those of the individual components, confirming reorganization of electron density distribution within both molecules. These spectral changes confirm that decanoic acid donates its carboxylic −OH proton to the carbonyl oxygen of anisaldehyde, establishing the O−H···O=C hydrogen-bonding network responsible for the eutectic behavior and the selective solvation properties exploited during DLLME extraction. These spectral changes reflect substantial alterations in the initial hydrogen-bonding network of the NADES (Figure 1).

### 2.2. UHPLC-MS/MS Method Optimization

The separation conditions were systematically adjusted to achieve superior sensitivity, selectivity, and chromatographic performance, while adhering to GAC guidelines. A short C18 column packed with 1.7 µm particles was selected to enable fast runs, minimize solvent consumption, and produce sharply defined peaks with high resolving power [34,35]. Ethanol was employed as an environmentally safer organic modifier, reinforcing the method’s eco-friendly profile.

**Figure 2:**
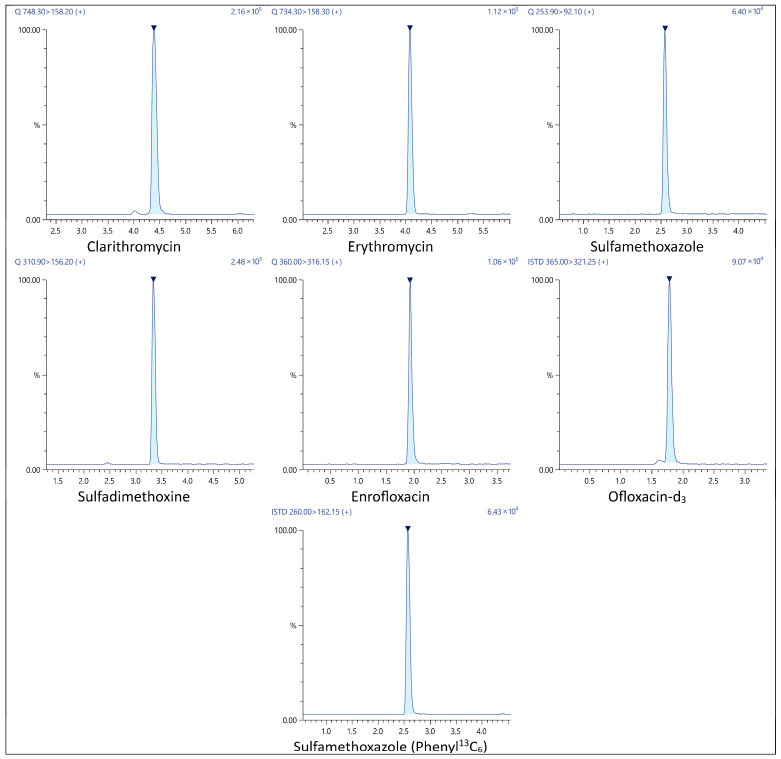
Representative LC–MS/MS chromatograms of target antibiotics using the developed NADES-DLLME-UHPLC–MS/MS method.

Multiple gradient elution programs were evaluated to achieve the best resolution within the shortest possible run time. Under the optimized conditions, all five target antibiotics were fully separated and eluted in 4 min (Figure 2). The analytes were quantified without interference from the NADES matrix. Although shorter retention times could be achieved, they led to co-elution with NADES constituents. To maintain the high sensitivity and selectivity necessary for trace-level determination of the target compounds in meat and poultry samples, tandem mass spectrometry (MS/MS) detection was employed.

### 2.3. Optimization of the NADES-DLLME Method

The NADES-based DLLME procedure was systematically optimized to achieve maximum extraction performance. A stepwise one-variable-at-a-time approach was employed to optimize key experimental conditions, including the selection of NADES, molar ratio and volume, sample volume, ionic strength, pH, vortexing mixing time, and centrifugation duration.

#### 2.3.1. NADES Type, Molar Ratio and Volume

**Figure 3:**
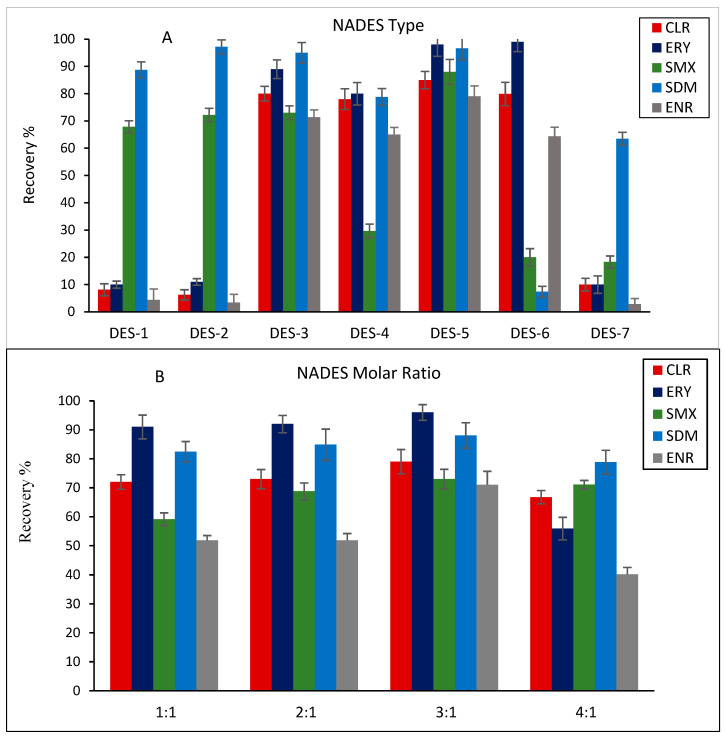
Effect of (**A**) NADES type and (**B**) NADES molar ratio on the extraction recovery of the studied analytes.

Different systems have been investigated including decanoic acid with formic acid (NADES-1), acetic acid (NADES-2), octanoic acid (NADES-3), oleic acid (NADES-4), decanoic acid (NADES-5), thymol (NADES-6), and fenchone (NADES-7). Among the tested NADES formulations, the combination of anisaldehyde and decanoic acid demonstrated the highest extraction efficiency for all target analytes. The recoveries obtained with the different NADES systems are illustrated in Figure 3A. This superior performance is likely due to strong interactions between the analytes and the solvent, including hydrogen bonding and dipole–dipole forces. Based on these results, the anisaldehyde–decanoic acid NADES was selected as the extraction medium for all subsequent analyses.

The molar ratio of the synthesized NADES is a key factor influencing the physicochemical characteristics of the NADES, which in turn affects its extraction performance. To identify the most effective ratio, several anisaldehyde–decanoic acid NADES formulations were tested at different molar proportions (1:1, 2:1, 3:1 and 4:1). As shown in Figure 3B, the 3:1 ratio delivered the highest extraction recovery for all target analytes and was therefore selected as the optimal composition for subsequent experiments.

**Figure 4:**
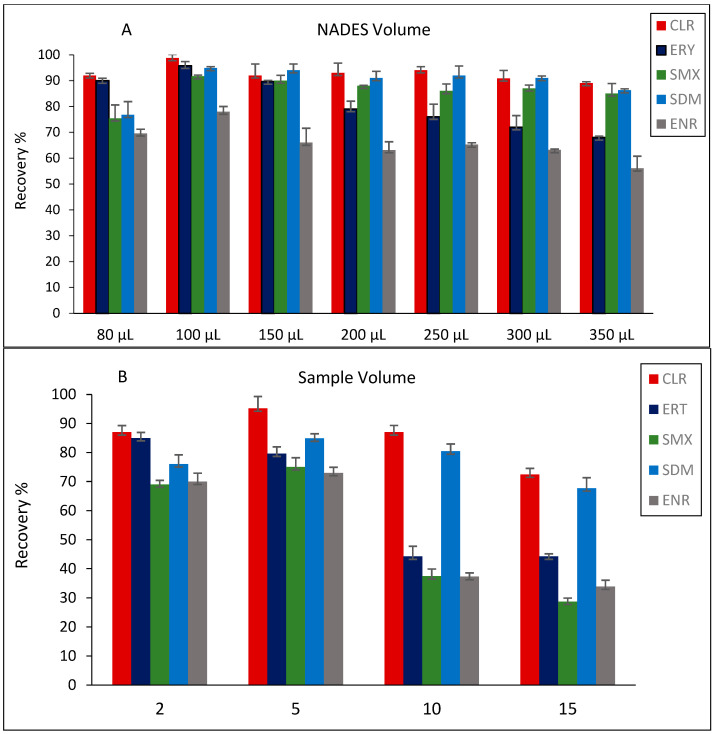
Effect of (**A**) NADES volume and (**B**) sample volume on the extraction recovery of the studied analytes.

Various volumes of anisaldehyde–decanoic acid NADES, ranging from 80 to 350 µL, were tested in triplicate to determine the optimum amount for efficient extraction. As shown in Figure 4A, increasing the volume from 80 μL to 100 μL led to a marked improvement in analyte recoveries. However, when the volume exceeded 100 μL, the extraction efficiency decreased, which may be attributed to dilution effects caused by excess solvent. Conversely, insufficient NADES volumes resulted in incomplete extraction and reduced recovery due to inadequate interaction with the analytes. Based on these results, 100 μL was selected for further experiments.

#### 2.3.2. Sample Volume

Sample volumes of 2, 5, 10, and 15 mL were examined to establish the most appropriate amount for effective extraction. As presented in Figure 4B, a decline in recovery was observed when the volume exceeded 5 mL, most likely due to a dilution effect that reduces the concentration of analytes available for extraction. In contrast, very small sample volumes did not provide sufficient analyte content for reliable detection. Based on these findings, 5 mL was selected as the optimal sample volume for the analysis of real samples.

#### 2.3.3. NaCl Concentration

**Figure 5:**
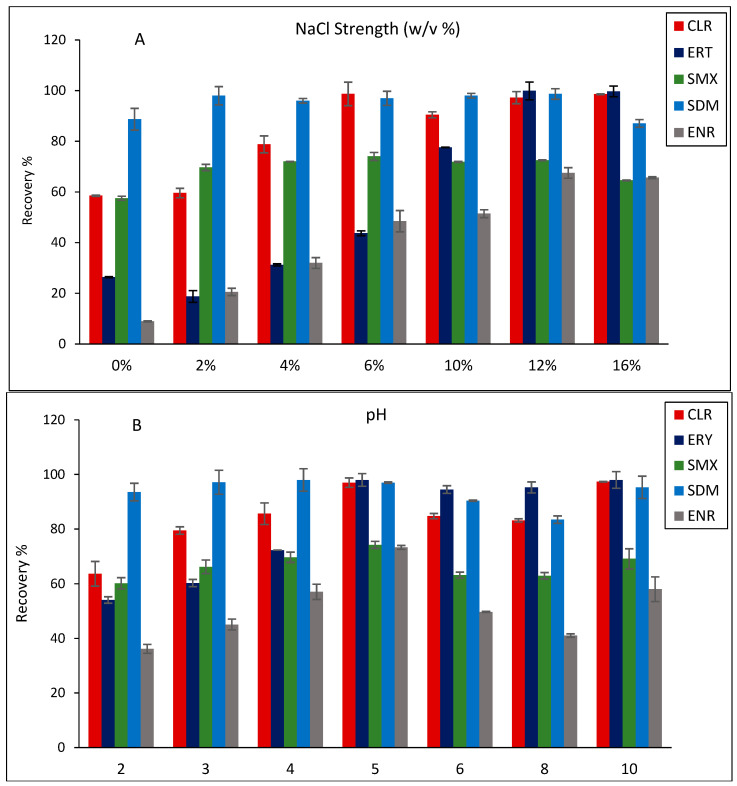
Effect of (**A**) NaCl strength and (**B**) pH on the extraction recovery of the studied analytes.

Conducting the microextraction without NaCl led to low extraction efficiencies for all analytes. Increasing the NaCl concentration from 0% to 12% enhanced the extraction recovery of all compounds. This improvement can be attributed to the salting-out effect, whereby the increased ionic strength reduces the solubility of the analytes in the aqueous phase and promotes their transfer into the NADES extractant phase. However, further increasing the salt concentration beyond 12% caused a decline in the recovery of SMX, SDM, and ENR (Figure 5A). This decrease may be associated with the elevated viscosity of the extraction medium and mass-transfer limitations at higher salt concentrations, which can hinder the efficient partitioning of some analytes into the extraction phase. Therefore, 12% NaCl (equivalent to 1200 mg per 10 mL sample) was selected as the optimal amount for all subsequent experiments.

#### 2.3.4. Sample pH

The influence of sample pH on extraction efficiency was investigated over the pH range of 2–10 (Figure 5B). Increasing the pH from two to five enhanced the extraction recovery for all analytes. This behavior can be attributed to changes in the ionization state of the target compounds, where pH five favors the presence of less ionized species with greater affinity for the NADES phase. However, when the pH exceeded five, the recovery of most analytes decreased, likely due to increased ionization, which enhances their solubility in the aqueous phase and reduces their partitioning into the extraction solvent. Therefore, pH five was selected as the optimal condition.

#### 2.3.5. Vortexing Time

**Figure 6:**
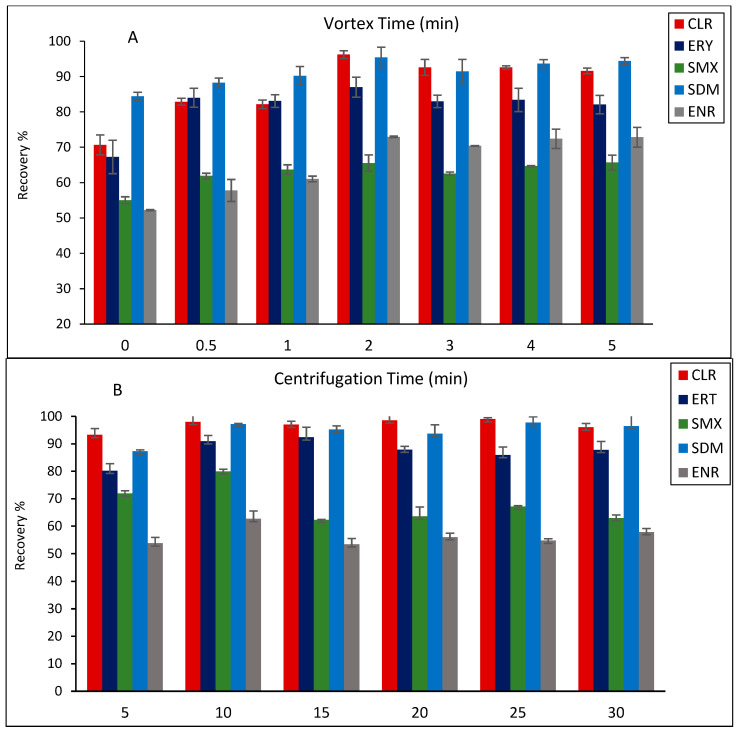
Effect of (**A**) vortex time and (**B**) centrifugation time on the extraction recovery of the studied analytes.

The influence of vortex agitation time on extraction efficiency was assessed over a range of 0 to 5 min. Extending the vortex duration enhanced the extraction recovery of all analytes (Figure 6A), as vigorous mixing promotes the dispersion of NADES into fine droplets with greater interfacial surface area, thereby improving mass transfer during extraction. However, the improvement in recovery became negligible beyond 2 min of vortexing. Therefore, a vortex time of 2 min was selected as the optimal condition.

#### 2.3.6. Centrifugation Time

The effect of centrifugation time on the extraction recovery of the target analytes was investigated by examining durations between 5 and 30 min at a fixed speed of 3500 rpm. Longer centrifugation facilitates the complete settling and aggregation of the finely dispersed NADES droplets into a recoverable phase suitable for subsequent analysis. As shown in the recovery data (Figure 6B), extending the centrifugation time from 5 to 10 min produced a marked improvement in extraction efficiency. Further increases beyond 10 min offered minimal additional benefit while increasing total analysis time. Consequently, a centrifugation duration of 10 min was chosen as the optimal condition.

### 2.4. Method Validation

Assessment of the method’s performance involved evaluating key analytical parameters such as linearity, accuracy, precision, and the detection and quantification limits.

Matrix-matched calibration was carried out using meat and chicken matrices that were verified to be free of the target analytes. Calibration curves were generated using seven concentration levels spanning 0.125–50 μg·kg^−1^ for processed meat and poultry products. The method demonstrated excellent linearity, with determination coefficients (R^2^) of ≥0.9982 for the meat-based samples and ≥0.9985 for the poultry-based samples (Table 1).

The limits of detection (LODs) and quantification (LOQs) were established using signal-to-noise ratios of 3:1 and 10:1, respectively. For meat products, LODs ranged from 0.003 to 0.023 μg·kg^−1^, while for poultry products they varied from 0.001 to 0.02 μg·kg^−1^, indicating the excellent sensitivity of the developed method for trace-level analysis in complex food matrices (Table 1). The precision of the method was evaluated in terms of intra-day (n = 6) and inter-day (n = 6) variability at three different concentration levels.

The relative standard deviations (RSDs) did not exceed 5.75% for meat products and 5.08% for poultry products, demonstrating strong repeatability and consistency of both the extraction and measurement procedures.

For assessing the method accuracy, recovery experiments were performed at three spiking levels, with recoveries ranging from 84.1% to 99.4%. These findings demonstrate the reliability of the proposed method for quantifying the target analytes in both meat and poultry matrices. Overall, the method exhibited excellent analytical characteristics, including high sensitivity, good precision, and satisfactory accuracy, making it appropriate for routine surveillance of antibiotic residues in food samples. A summary of the main analytical parameters is presented in Table 2.

**Figure 7:**
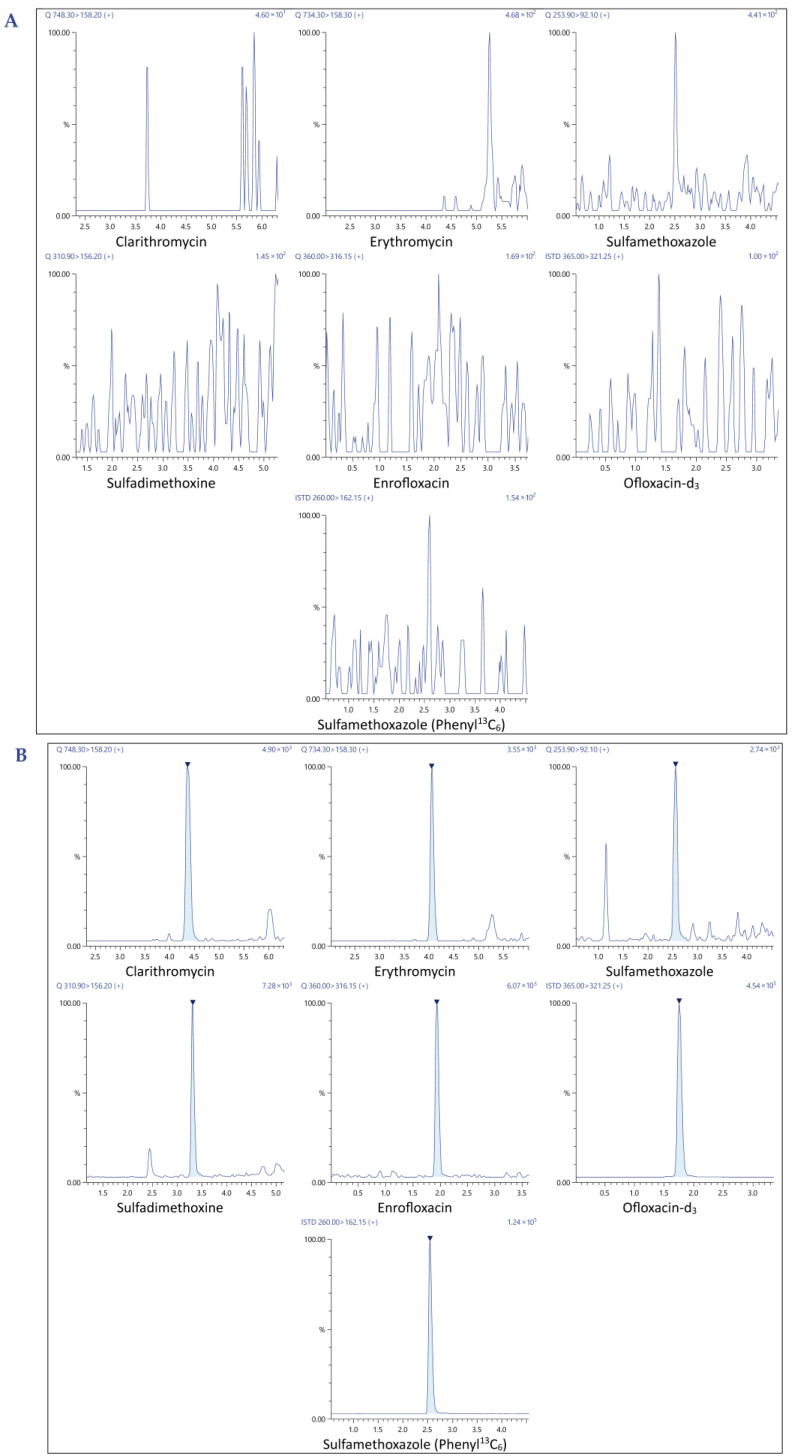
Representative chromatograms obtained using the developed NADES-DLLME–UHPLC–MS/MS method: (**A**) blank matrix and (**B**) spiked sample.

Matrix effects are critical validation parameters in LC-MS/MS food analysis, as co-eluting endogenous matrix components compete with target analytes for ionization in the ESI source, causing suppression or enhancement that compromises quantitative accuracy [36]. ME values for all five antibiotics in both matrices are summarized in Table 2. All values remained within the ±20% threshold, confirming the absence of significant matrix-related bias [36]. Clarithromycin and erythromycin exhibited slight ion enhancement in both matrices (CLR: +13.5% meat, +8.3% chicken; ERY: +11.1% meat, +13.8% chicken), consistent with published observations for hydrophobic macrolides under positive ESI conditions, where trace polar co-extractables promote gas-phase protonation of basic nitrogen-containing analytes [37]. Conversely, SMX, SDM, and ENR showed ion suppression across both matrices (SMX: −16.6% meat, −18.2% chicken; SDM: −8.8% meat, −9.8% chicken; ENR: −13.2% meat, −12.5% chicken), in agreement with the literature [37,38]. This is attributed to the selective solvation properties of the anisaldehyde–decanoic acid NADES, which preferentially partitions the target analytes while excluding polar water-soluble co-extractables that normally drive ESI enhancement. The consistent moderate suppression of ENR in both matrices is further explained by the well-established chelation properties of the fluoroquinolone 4-oxo-3-carboxyl pharmacophore, which forms stable bidentate complexes with divalent and trivalent metal ions (Mg^2+^, Ca^2+^, Fe^3+^) abundantly present in meat and poultry tissues [39]. These metal–drug complexes, co-partitioned into the extract, compete with the free protonated analyte at the ESI droplet surface, reducing ionization efficiency, which is a matrix-dependent suppression pattern consistently reported for fluoroquinolones in animal-derived food matrices [40]. Overall, the NADES-DLLME procedure yielded substantially cleaner extracts compared to conventional techniques, with all ME values within regulatory acceptance criteria. Residual matrix effects were further compensated by matrix-matched calibration and isotopically labeled internal standards (OFX-d_3_ for ENR and SMX-phenyl-^13^C_6_ for all remaining analytes). A blank matrix chromatogram, demonstrating the absence of interfering peaks at the retention times of the target analytes, and a chromatogram of a spiked sample, confirming their successful detection, are shown in Figure 7.

### 2.5. Real Sample Analysis

**Figure 8:**
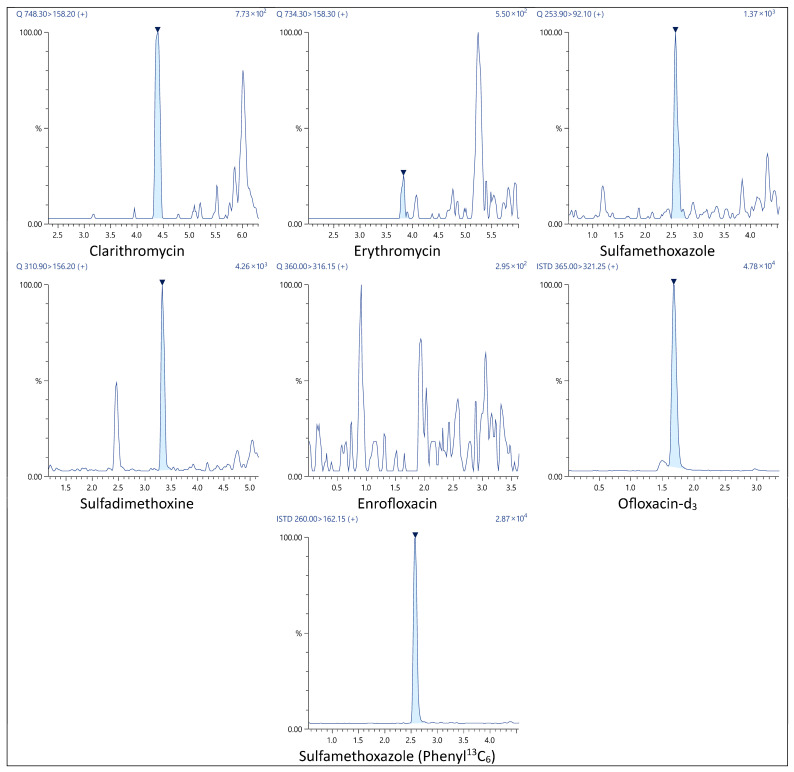
Representative chromatogram of Sample No. 5 obtained using the proposed method.

A total of 40 processed meat and frozen poultry products collected from the Saudi market were analyzed for five commonly used veterinary antibiotics (Table 3). The results revealed substantial variation in both the frequency and concentration of residues across product types. SMX was the most frequently detected analyte, occurring in 37 samples (92.5%) with concentrations ranging from 0.61 to 37.7 μg·kg^−1^. SDM was similarly widespread, identified in 34 samples (85.0%) at levels up to 53 μg·kg^−1^. A representative chromatogram of a real sample is presented in Figure 8. The high prevalence of sulfonamides in the analyzed samples indicates extensive use of these drugs in livestock production. Although the detected concentrations were below commonly adopted maximum residue limits (MRLs) for sulfonamides (100 μg·kg^−1^ for muscle tissue [7,41]), their ubiquitous occurrence suggests continuous consumer exposure and highlights the potential risk of contributing to sulfonamide-resistant bacterial populations.

CLR was detected in 28 samples (70.0%). Although the majority of samples contained low-to-moderate levels, three poultry samples showed exceptionally high concentrations, reaching up to 749.28 μg·kg^−1^. Since CLR lacks established MRLs in many livestock species, the detected residues pose an important safety issue. The relatively high concentration of CLR detected in some poultry samples raises concerns regarding non-compliant, potential off-label or unregulated use in poultry production. These findings emphasize the need for strict enforcement of withdrawal periods and strengthened monitoring programs to ensure compliance with good veterinary practices and to protect consumer health. The high concentrations observed reinforce the necessity for regulatory agencies to introduce specific limits and increase monitoring of macrolide use, especially in poultry. ENR was found in 25 samples (62.5%), with concentrations ranging from 0.38 to 32.73 μg·kg^−1^. Although its measured levels were below the commonly established MRL for muscle tissue (100 μg·kg^−1^), the frequent occurrence of this fluoroquinolone is noteworthy due to its critical role in both veterinary and human medicine. ERY exhibited the lowest detection frequency, appearing in 14 samples (40.0%) with a maximum concentration of 14.7 μg·kg^−1^, far below its MRL of 200 μg·kg^−1^ [41]. Although ERY concentrations are well-below regulatory limits, its co-occurrence with other antibiotics highlights the need to monitor the use of all macrolides. The frequent detection of antibiotics underscores significant concerns for antimicrobial stewardship and food safety practices. These results emphasize the need for ongoing monitoring of drug residues. Enhancing regulatory oversight and encouraging responsible use of antibiotics in livestock production are crucial steps to reduce consumer exposure and limit the development and spread of antimicrobial resistance.

### 2.6. Comprehensive Assessment of the Method Performance

The proposed method was thoroughly assessed for its environmental friendliness, practical usefulness and innovation. A set of eight complementary metrics was employed to capture these aspects from multiple perspectives. By combining these different evaluation tools, a comprehensive and well-rounded characterization of the method was achieved, demonstrating its consistency with contemporary principles of green and sustainable analytical science.

To obtain a thorough evaluation of the greenness characteristics of the developed method, five established assessment approaches were employed to examine its environmental footprint, safety aspects, and possible effects on human health. The application of these complementary tools offers a comprehensive, multidimensional insight into the method’s conformity with the principles of GAC.

**Figure 9:**
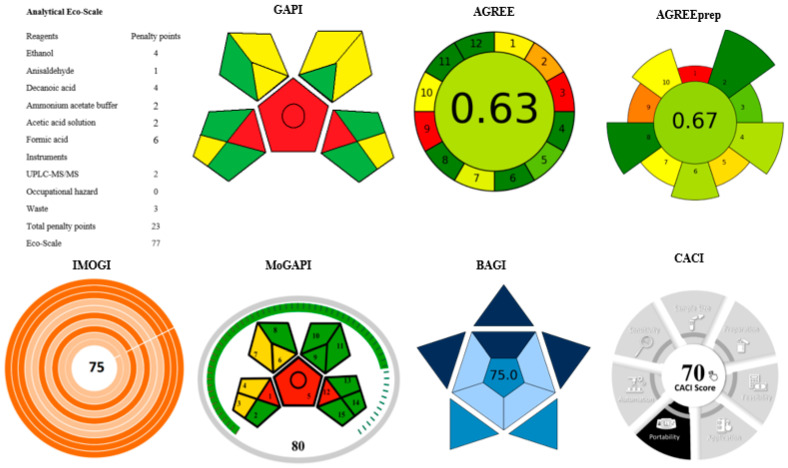
Assessment of the performance of the developed method using Analytical Eco-Scale, GAPI, AGREE, AGREEprep, MoGAPI, BAGI, CACI and IMOGI tools.

The Analytical Eco-Scale (AES) [26] was applied as part of the evaluation. This approach allocates penalty points according to several factors, including the nature and amount of the reagents, occupational and safety risks, waste production, and energy requirements. The Eco-Scale score is calculated by subtracting the accumulated penalty points from a theoretical maximum of 100. In this evaluation framework, values exceeding 75 indicate excellent green performance, scores ranging from 50 to 75 are regarded as acceptable, while values below 50 reflect poor greenness. In the present work, an AES value of 77 places the method in the excellent green category. This favorable score is attributed to the use of a natural deep eutectic solvent (anisaldehyde–decanoic acid), reduced consumption of chemicals, and minimal waste production (Figure 9).

The Green Analytical Procedure Index (GAPI) [27] was also employed, providing a comprehensive visual overview of the method’s environmental performance throughout the entire analytical process. GAPI is displayed as a five-section pictogram, with each section representing a distinct stage of the procedure. These sections are color-coded to indicate environmental impact: green for low, yellow for moderate, and red for high impact. A greater proportion of green areas signifies improved environmental performance. Detailed information on the construction and interpretation of GAPI can be found in reference [27]. In the case of the proposed method, the GAPI evaluation revealed six green zones, reflecting minimal environmental impact in key stages such as sample preparation, solvent consumption, and waste management. Only three red zones were observed, which were mainly attributed to the use of MS/MS detection. This technique is unavoidable to achieve the high sensitivity and selectivity required for the determination of antibiotic residues in food matrices. In addition, one red zone was related to the inclusion of a sample preparation step, which is essential for analyte preconcentration (Figure 9).

The Analytical GREEnness Metric (AGREE) [28] complements the assessment by presenting the evaluation in a circular diagram divided into twelve segments, each representing one principle of GAC. Each segment is color-coded along a gradient from red to green, indicating the degree of compliance with the respective principle, while the central value summarizes the overall greenness score on a scale ranging from 0 (least green) to 1 (most green). This approach enables AGREE to deliver both numerical and visual interpretation of the method’s environmental impact [28]. In this study, the AGREE score of 0.63 (Figure 9) reflects an acceptable level of adherence to GAC principles, largely attributed to the application of renewable solvent systems and the reduced use of samples and reagents.

The Analytical GREEnness Metric for Sample Preparation (AGREEprep) [29] is a dedicated metric developed to assess the environmental sustainability of sample preparation steps in analytical workflows. It builds on the concept of the original AGREE approach by concentrating specifically on the pre-analytical phase, which is often responsible for most solvent use, waste production, and energy consumption. The AGREEprep tool evaluates the greenness of sample preparation based on clearly defined criteria derived from the ten principles of green sample preparation. These criteria account for parameters such as sample size, the nature and quantity of solvents, energy demand, degree of automation, waste handling, and user safety. The output is presented as a color-coded visual score on a scale from 0 to 1, representing the overall sustainability of the sample preparation procedure. In the present study, an AGREEprep value of 0.67 reflects a high degree of greenness for the proposed method (Figure 9).

To enhance the robustness of the assessment, the Modified Green Analytical Procedure Index (MoGAPI) [30] was additionally employed. In contrast to the conventional GAPI approach, MoGAPI applies a more refined scoring scheme that quantitatively expresses the level of greenness by coupling numerical values with the visual pictogram. This refinement enables clearer discrimination among methods that may display similar GAPI profiles, making MoGAPI particularly valuable for comparative evaluations. In this study, the MoGAPI analysis yielded a score of 80 (Figure 9), highlighting low chemical risk and a reduced environmental impact.

To assess the method’s practical applicability and its feasibility for real-world use, the Blue Assessment of Green Index (BAGI) [31] and the Click Analytical Chemistry Index (CACI) [32] were employed. BAGI is a recently introduced evaluation framework that broadens sustainability assessment beyond environmental considerations by incorporating economic viability and social impact. By combining these dimensions, BAGI offers an integrated perspective on the overall sustainability performance of analytical methodologies. The proposed method attained a BAGI score of 75, reflecting a high level of compliance with the principles of sustainable analytical chemistry (Figure 9) and demonstrating advantages that support both environmentally responsible practices and efficient analytical workflows.

CACI is an intuitive evaluation framework developed to examine the feasibility and practical applicability of analytical methods. It provides a digital interface that enables the comparison of different methodologies in terms of sustainability, usability, and overall performance. By placing strong emphasis on innovation and future readiness, the CACI approach helps ensure that newly developed methods satisfy both academic and industrial requirements [32]. In this work, the proposed method achieved a CACI score of 70, reflecting good performance and a high level of practical suitability (Figure 9).

For evaluating the innovation level of the developed method, the recently introduced Innovative Method Orange Gradient Index (IMOGI) was used [33]. IMOGI is a recently developed tool designed to assess analytical methods in terms of innovation. It is based on ten established criteria, including different aspects such as originality, technological progress, automation, method miniaturization, and sustainability principles, to provide a transparent and reproducible assessment. The results are displayed as a color-coded concentric orange gradient chart with a cumulative score (0–100), enabling rapid benchmarking and clear differentiation between highly innovative, acceptable, and low-innovation methods [33]. The developed method achieved an IMOGI score of 75 (Figure 9), reflecting a strong degree of methodological innovation. This performance is attributed to the combination of novel solvent design with a miniaturized, efficient extraction strategy alongside comprehensive greenness assessment. Overall, the results emphasize the ongoing transition in analytical chemistry toward more sustainable and high-performance analytical approaches. This strategy illustrates how modernization in analytical chemistry can arise from combining natural product-based materials, methodological simplification, and sustainability-driven design.

In summary, the developed method shows excellent environmental and analytical performance, driven by sustainable sample preparation using natural deep eutectic solvents and microextraction. Comprehensive evaluation confirmed it as a safe, cost-effective, environmentally benign, and safe alternative to traditional analytical methods. It is consistent with the principles of modern green and white analytical chemistry and is well suited for applications in food safety surveillance.

### 2.7. Comparison to Other Reported Methods

A variety of analytical approaches have been previously described for the determination of antibiotic residues in food matrices. When compared with existing methodologies, the proposed anisaldehyde–decanoic acid NADES-based DLLME procedure combined with UHPLC–MS/MS demonstrates markedly superior sensitivity, as reflected by its substantially lower limits of detection. The method also shows excellent analytical performance, including high recoveries, strong linearity and precision, with results that are comparable to or exceed those obtained using conventional extraction strategies such as solid-phase extraction.

In addition to its analytical advantages, the combination of NADES-based DLLME with UHPLC–MS/MS offers improved chromatographic performance along with a markedly better environmental profile when compared to previously reported approaches (Table 4). In contrast to conventional methods that depend on hazardous, toxic, or poorly degradable organic solvents, the present strategy utilizes greener constituents such as water, ethanol, and anisaldehyde, thereby minimizing environmental impact. The extraction process is rapid, consumes only small solvent volumes, and eliminates the need for extensive cleanup procedures, which results in shorter run times and increased sample throughput while ensuring accurate quantification in complex processed meat and poultry matrices.

Overall, compared with existing methods, the proposed procedure is simpler, faster, more sensitive, more economical, and environmentally safer, while maintaining high analytical reliability. Accordingly, the integration of NADES-DLLME with UHPLC–MS/MS provides a sustainable and efficient alternative for routine determination of antibiotic residues in processed meat and poultry products.

## 3. Materials and Methods

### 3.1. Materials

Clarithromycin (CLR, >99.0%) was obtained from UFC Biotechnology (Buffalo, NY, USA), and erythromycin (ERY), sulfamethoxazole (SMX), sulfadimethoxine (SDM), enrofloxacin (ENR), ofloxacin-d_3_ (OFX-d_3_) and sulfamethoxazole (phenyl−^13^C_6_) (SMX−^13^C_6_), each with a purity of ≥98%, were obtained from Sigma (Steinheim, Germany). The selection of these two isotopically labeled internal standards was based on structural analogy, chromatographic behavior, and practical availability. OFX-d_3_, a deuterium-labeled fluoroquinolone sharing the core 4-oxo-quinoline-3-carboxylic acid pharmacophore with enrofloxacin, ensures near-identical extraction recovery and ESI response, providing reliable compensation for matrix effects affecting the fluoroquinolone class [47]. SMX-^13^C_6_ corrects for matrix effects and extraction variability affecting both sulfamethoxazole and sulfadimethoxine, which share the same sulfonamide pharmacophore and closely similar chromatographic elution behavior [48]. For clarithromycin and erythromycin, SMX-^13^C_6_ was applied as a surrogate internal standard, as the scope of this study prioritized class-representative IS coverage using two well-characterized stable isotope standards. This approach is permitted when class-matched IS are unavailable, provided acceptable analytical performance is demonstrated [49], and was empirically confirmed by the acceptable ME values (CLR: +13.5% meat, +8.3% chicken; ERY: +11.1% meat, +13.8% chicken) and high recoveries (84.1–99.4%) obtained for both macrolides.

Formic acid, octanoic acid, decanoic acid, thymol, fenchone, anisaldehyde and ammonium acetate (LC-MS grade; purity ≥ 98%), acetic acid (≥ 99.7%), and oleic acid (≥99%) were obtained from Sigma (Steinheim, Germany).

Stock solutions of CLR, ERY, SMX, SDM and ENR were prepared in ethanol (1000 mg L^−1^) and stored at −20 °C. Working solutions were obtained by appropriate dilutions in water. Solutions of isotopically labeled internal standards (OFX-d_3_ and SMX-^13^C_6_) were prepared by individually weighing 5 mg of each compound and dissolving them in 5 mL of ethanol. The resulting stock solutions were kept under frozen storage at −20 °C until further use. An internal standard (IS) solution mixture was prepared at a concentration of 500 ng·mL^−1^ of each IS through proper dilution in water and was subsequently used for IS calibration. LC-MS-grade ethanol was obtained from Merck (Darmstadt, Germany). Pure Lab Ultra water system from ELGA (High Wycombe, UK) was employed to prepare the ultrapure water.

### 3.2. Instrumentation

The analysis was conducted using Nexera X2 ultra-high-performance liquid chromatography system (Shimadzu, Japan) hyphenated to an 8050 triple quadrupole mass spectrometer (Shimadzu, Japan) operating with an electrospray ionization (ESI) interface. Instrument control, data acquisition, and processing were performed using LabSolution^®^ version 5.93 software. Chromatographic separation was carried out on a BEH C18 analytical column (100 × 2.1 mm, 1.7 µm; Waters, Milford, MA, USA) protected by a VanGuardguard column. Throughout the analysis, the column oven was maintained at 40 °C, while the mobile phase was delivered at 0.3 mL min^−1^.

The separation employed a binary gradient system composed of water containing 0.1% formic acid (mobile phase A) and ethanol (mobile phase B). Aliquots of 2 µL were injected into the system. The gradient program commenced with 15% B, increased to 30% B after 1.5 min, and then progressively rose to 100% B by 4 min. Subsequently, the initial mobile-phase composition was restored within 0.5 min and maintained for an additional 2 min to allow column reconditioning before the next injection.

For the mass spectrometric measurements, the system operated in positive-ion mode using multiple reaction monitoring (MRM). The precursor–product ion transitions for each target compound were established through flow injection experiments combined with the automated MRM optimization function available in LabSolutions^®^. Detailed MRM optimized parameters are presented in Table 5. The ESI interface operated at 300 °C, with an interface voltage of 4.0 kV. The desolvation line was operated at 250 °C, whereas the heat block temperature was adjusted to 400 °C. Compressed air served as the heating gas at a flow of 10 L min^−1^. Nitrogen was employed for nebulization and drying at flow rates of 3 and 10 L min^−1^, respectively. Argon was used as the collision-induced dissociation (CID) gas. Per-compound collision energies and MRM transitions are listed in Table 5. Analyte confirmation was achieved by matching both the chromatographic retention time and the relative intensities of two selected MRM transitions with those obtained from reference standards, allowing a maximum deviation of 20%. Quantitative analysis was subsequently carried out using the MRM transition that produced the highest signal intensity for each confirmed analyte.

Infrared spectra were recorded using the attenuated total reflection (ATR) technique on a Nicolet 50 Fourier transform infrared (FTIR) spectrometer (Thermo Scientific^®^, Waltham, MA, USA). Proton nuclear magnetic resonance (^1^HNMR) measurements were carried out at ambient temperature on a 300 MHz Bruker NMR instrument (Bruker, Fällanden, Switzerland). Samples were diluted in deuterated dimethyl sulfoxide (DMSO-d_6_) and transferred into standard 5 mm NMR tubes prior to analysis.

### 3.3. Sample Collection and Preparation

Processed meat products (sausages, turkey slices, and roast beef; n = 20) and frozen poultry items (chicken tenders, thighs, and drumsticks; n = 20) were randomly purchased from supermarkets in Al Khobar and Dammam, Saudi Arabia. All samples were transported to the laboratory in insulated ice boxes, coded upon arrival, minced, and thoroughly homogenized.

For extraction, 2.0 g of homogenized sample was placed in a 50 mL polypropylene centrifuge tube, followed by the addition of 250 µL of an internal standard solution (1000 µg·L^−1^). Next, 2 mL of 0.6% acetic acid was added, and the mixture was agitated for 1 min to ensure proper dispersion. Thereafter, 4 mL of ethanol was introduced, and mixing was continued for an additional 2 min. The samples were then centrifuged at 3500 rpm for 10 min. Following centrifugation, the clear extract was collected and transferred to a 25 mL volumetric flask. The solution was diluted to volume with 0.2 M ammonium acetate buffer adjusted to pH 5 and thoroughly mixed using a vortex mixer. The resulting extract was subsequently used for the DLLME step.

### 3.4. Preparation of NADES

To determine the most effective NADES formulation for the extraction process, seven binary solvent systems were prepared and tested. Anisaldehyde was combined separately with different hydrogen donors, yielding the following NADES compositions: formic acid (NADES-1), acetic acid (NADES-2), octanoic acid (NADES-3), oleic acid (NADES-4), decanoic acid (NADES-5), thymol (NADES-6), and fenchone (NADES-7). Each pair of components was mixed at a 1:1 molar ratio and heated to 70 °C with continuous stirring until a transparent and uniform liquid was obtained. After preparation, the NADES mixtures were stored in a desiccator to minimize moisture uptake and maintain its physicochemical properties. No phase separation or noticeable changes were observed during the course of the experiments.

### 3.5. DLLME Procedure

An aliquot of 5 mL of the sample solution was accurately transferred into a 15 mL polypropylene centrifuge tube. Subsequently, 1200 mg of sodium chloride was introduced and vortex-mixed until complete dissolution was achieved. Following this, 100 µL of anisaldehyde–decanoic acid NADES with a molar ratio of 3:1 was added, and the solution was vortexed for 2 min until a cloudy solution was formed. The resultant mixture was centrifuged for 10 min at 3500 rpm. Subsequently, the aqueous phase was discarded using a syringe. The collected organic phase was subsequently combined with 100 µL ethanol and homogenized by thorough mixing. The mixture was then filtered by 0.2 µm nylon nano-filter vials from (Restek^®^, Bellefonte, PA, USA). After filtration, an aliquot of 2 µL was introduced into the LC-MS/MS system for chromatographic analysis.

### 3.6. Method Validation

Validation of the NADES-DLLME-UHPLC-MS/MS method was carried out by examining linearity, accuracy, precision, limits of detection (LODs), and limits of quantitation (LOQs). Matrix effects were investigated using blank meat and poultry samples confirmed to be free of the target antibiotics. These blanks were fortified at three concentration levels (0.25, 12.5 and 50 µg·kg^−1^) to assess both accuracy and precision, with six independent replicates prepared and analyzed at each level. Method validation was performed in accordance with the guidance document on analytical quality control and method validation procedures for residues analysis in food and feed (SANTE/11312/2021, Version 2, European Commission, 2023) [49].

To maintain analytical reliability, a procedural blank consisting solely of ultrapure water was injected after every ten samples to ensure that no contamination occurred throughout the run. Calibration curves specific to each matrix were generated by spiking homogenized blank samples with eight different concentrations of the analytes, followed by extraction using the optimized procedure. Linearity was evaluated by calculating the slope, intercept, and correlation coefficient for each calibration curve. Limits of detection and quantification were derived from signal-to-noise ratios of 3:1 and 10:1, respectively.

Accuracy was expressed as the percentage of analyte recovered from spiked blank samples, which were prepared in triplicate at the three validation concentrations. Precision was assessed through both repeatability and intermediate precision studies. Repeatability (intra-day precision) was determined from six replicate measurements performed on the same day, whereas intermediate precision (inter-day precision) involved analyzing the fortified samples over three consecutive days. For both, the relative standard deviation (%RSD) was calculated for each target analyte in both matrices.

Matrix effects (MEs) were evaluated using the post-extraction spiking approach [50]. Homogenized blank meat and poultry samples of the same matrix type were used to evaluate ME. When endogenous residues of a target analyte were detected in the blank extract, the corresponding background signal was subtracted prior to ME calculation. This approach ensured accurate assessment of matrix-induced ionization bias, with internal standard (IS) normalization applied throughout [Matuszewski and ME-AM mine]. Three independent sets of solutions were prepared at three concentration levels: (i) a solvent set, consisting of standard solutions of the target analytes and IS prepared in mobile phase without matrix; (ii) a post-extraction spiked set, prepared by spiking homogenized matrix samples with known concentrations of the target analytes and IS, followed by the full NADES-DLLME extraction procedure; and (iii) a background set, consisting of unspiked matrix extracts processed identically to quantify endogenous analyte signals. The IS-normalized peak area ratios (analyte peak area/IS peak area) were calculated for each set, and ME was computed according to the following equation:

$$ME\left(\%\right)=\left(\frac{{\left(peak\;area\;ratio\right)}_{Post-extraction}-{\left(Peak\;area\;ratio\right)}_{Background}}{{\left(Peak\;area\;ratio\right)}_{Solvent}}-1\right)\times 100$$

where an ME value of zero indicates the complete absence of matrix-related ionization bias, negative values indicate ion suppression, and positive values indicate ion enhancement. ME values were within ±20% [36]. ME experiments were performed independently for both processed meat and frozen poultry matrices, with six replicates at each concentration level.

## 4. Conclusions

A rapid, highly sensitive, and sustainable analytical method was established and validated in this study for the simultaneous determination of selected antibiotic residues in processed meat and poultry products obtained from the Saudi market. The proposed approach integrates NADES-based dispersive liquid–liquid microextraction with UHPLC–MS/MS analysis. The method demonstrated strong analytical performance, with good linearity (*r*^2^ ≥ 0.9982), low detection limits (0.001–0.023 μg·kg^−1^), high recovery rates (84.1–99.4%), and satisfactory precision (RSDs ≤ 5.75%). Sulfamethoxazole was detected most frequently, followed by sulfadimethoxine and clarithromycin. The method aligns with GAC principles through the use of a NADES composed of anisaldehyde and decanoic acid, which eliminates hazardous organic solvents and reduces waste generation. Comprehensive evaluation using eight assessment metrics confirmed its environmental friendliness, sustainability, and practical applicability. Overall, the method is suitable for routine food safety monitoring and supports sustainable analytical practices.

The method is limited by its focus on a restricted number of antibiotic classes, which may not fully represent the diversity of veterinary drug residues that can occur in food matrices. Future work will therefore aim to expand the analytical scope to include additional antibiotic families, enabling more comprehensive multi-residue screening. Further development will also focus on applying the method to a broader range of food matrices with varying complexity in order to evaluate its robustness and general applicability under diverse analytical conditions. In addition, future investigations will include detailed dietary exposure assessment and health risk characterization based on the measured residue levels, to better understand potential consumer exposure and associated public health implications.

## Figures and Tables

**Table 1:** Analytical performance of the developed NADES-DLLME-UHPLC method.

Matrix	Analytes	Linearity Range *	Regression Equation (n = 8)		Determination Coefficient (*r*^2^)	LOD *	LOQ *
			Slop	Intercept			
Processed meat products	CLR	0.125–50	47.8 × 10^−2^	4.13 × 10^−2^	0.9996	0.003	0.008
	ERY	0.125–50	9.86 × 10^−2^	2.30 × 10^−2^	0.9982	0.007	0.022
	SMX	0.125–50	9.94 × 10^−2^	2.94 × 10^−2^	0.9995	0.023	0.068
	SDM	0.125–50	35.9 × 10^−2^	3.76 × 10^−2^	0.9993	0.014	0.042
	ENR	0.125–50	12.1 × 10^−2^	3.83 × 10^−2^	0.9996	0.011	0.033
Frozen poultry products	CLR	0.125–50	50.1 × 10^−2^	4.71 × 10^−2^	0.9999	0.001	0.004
	ERY	0.125–50	16.2 × 10^−2^	1.60 × 10^−3^	0.9985	0.007	0.022
	SMX	0.125–50	10.8 × 10^−2^	1.51 × 10^−2^	0.9999	0.020	0.062
	SDM	0.125–50	39.0 × 10^−2^	2.88 × 10^−2^	0.9995	0.012	0.035
	ENR	0.125–50	13.9 × 10^−2^	−1.67 × 10^−2^	0.9997	0.009	0.028

* (μg·kg^−1^).

**Table 2:** Accuracy and precision of the developed NADES-DLLME-UHPLC method.

Matrix	Analytes	Intra-Day Precision						Inter-Day Precision						Matrix Effect * (%)
		High		Medium		Low		High		Medium		Low		
		% R	%RSD	% R	%RSD	% R	%RSD	% R	%RSD	% R	%RSD	% R	%RSD	
Processed meat products	CLR	93.3	3.30	98.5	3.28	99.4	3.18	94.1	2.90	97.8	2.83	98.0	3.53	+13.5
	ERY	88.4	4.51	86.8	3.02	88.7	4.17	86.7	4.83	84.8	2.77	87.2	4.90	+11.1
	SMX	84.6	4.21	89.1	5.37	84.1	3.33	84.9	3.41	87.3	5.75	85.4	3.21	−16.6
	SDM	88.5	3.23	90.5	4.93	97.8	3.90	89.5	3.82	89.8	4.50	95.9	4.67	−8.8
	ENR	84.2	4.40	85.0	3.74	89.5	4.13	89.3	4.48	84.9	4.08	86.1	3.38	−13.2
														
Frozen poultry products	CLR	94.4	3.22	90.8	1.94	93.2	2.55	93.2	3.68	95.3	2.26	89.7	3.44	+8.3
	ERY	88.0	2.33	86.0	1.64	85.7	2.17	87.2	3.16	82.9	2.47	85.8	1.91	+13.8
	SMX	85.4	3.88	84.5	3.80	84.7	2.09	86.9	3.85	86.2	4.28	84.4	2.38	−18.2
	SDM	92.3	4.26	97.5	3.59	96.6	4.60	92.2	3.49	97.2	3.31	95.9	4.20	−9.8
	ENR	84.4	1.64	89.0	1.61	85.5	4.09	88.5	3.92	84.8	3.39	90.0	5.08	−12.5

High, medium and low concentrations are 0.25, 12.5 and 50 µg·kg^−1^, respectively. * ME (%) = (((peak area ratio)_Post−extraction_ − (Peak area ratio)_Background_)/(Peak area ratio)_Solvent_ − 1) × 100.

**Table 3:** Analysis of the investigated antibiotics in the analyzed food samples (μg·kg^−1^).

Sample No.	Type	CLR	ERY	SMX	SDM	ENR
1	Turkey slices	nd	14.33	10.69	nd	3.87
2	Sausages	nd	14.7	12.73	nd	4.94
3	Sausages	nd	0.26	1.44	nd	0.75
4	Roast beef	nd	nd	nd	0.79	nd
5	Sausages	40.28	13.74	28.57	29.46	nd
6	Roast beef	38.17	14.24	23.98	27.14	nd
7	Turkey slices	1.49	0.35	3.25	1.64	nd
8	Sausages	37.38	14.62	17.42	21.27	1.69
9	Roast beef	37.45	14.13	23.63	22.68	1.15
10	Turkey slices	0.05	0.35	4.39	0.99	0.38
11	Turkey slices	36.74	nd	16.52	18.5	10.45
12	Roast beef	36.32	nd	18.24	18.73	9.1
13	Roast beef	0.29	nd	1.22	0.16	0.96
14	Roast beef	37.35	nd	17.14	20.61	6.8
15	Turkey slices	37.43	nd	17.84	17.92	4.99
16	Sausages	40.42	nd	36.91	53	30.28
17	Turkey slices	40.83	nd	37.7	46.17	32.73
18	Roast beef	37.41	14.37	20.53	21.98	1.42
19	Sausages	nd	10.25	15.94	5.89	7.75
20	Sausages	nd	7.92	11.11	10.03	4.59
21	Chicken tenders	749.28	nd	10.58	6.23	nd
22	Chicken tenders	715.3	nd	9.29	5.33	nd
23	Chicken tenders	24.03	nd	0.91	0.64	0.85
24	Chicken tenders	37.58	nd	12.79	8.36	nd
25	Chicken tenders	37.82	nd	11.93	6.36	nd
26	Chicken tenders	35.57	nd	11.81	7.38	nd
27	Chicken tenders	35.98	nd	10.55	7.53	nd
28	Chicken thighs	35.77	nd	11.18	7.46	nd
29	Chicken thighs	0.3	nd	0.89	0.11	1.67
30	Chicken thighs	732.29	nd	9.94	5.78	nd
31	Chicken thighs	37.7	nd	12.36	7.36	nd
32	Chicken thighs	0.17	2.25	0.61	1.41	3.48
33	Chicken thighs	nd	nd	2.28	3.89	4.58
34	Chicken thighs	nd	3.36	0.97	nd	nd
35	Chicken drumsticks	28.92	1.89	7.69	2.37	4.59
36	Chicken drumsticks	nd	nd	3.22	4.47	5.99
37	Chicken drumsticks	21.54	5.86	1.09	nd	2.99
38	Chicken drumsticks	nd	nd	6.59	3.55	0.92
39	Chicken drumsticks	nd	nd	nd	nd	nd
40	Chicken drumsticks	nd	nd	nd	4.94	3.29
	Concentration range	0.05–749.28	0.26–14.7	0.61–37.7	0.11–53	0.38–32.73
	Average	104.07	8.29	12.00	11.77	6.01
	Frequency (%)	70.0	40.0	92.5	85.0	62.5

nd: Not detected.

**Table 4:** Comparison of the proposed NADES-DLLME-UHPLC method with other reported methods for determination of antibiotics in processed meat and chicken samples.

Matrix	Analytes	Separation Technique	Extraction Method	Solvents	Linearity Range	R^2^	LOD	Recovery	RSD	Ref.
Chicken tissues	Sulfamethoxazole, ciprofloxacin, erythromycin amoxicillin, ampicillin, penicillin, gentamicin	LC-UV	Solvent extraction	Acetonitrile and methanol	0.098–0.255 μg·kg^−1^	≥0.9983	0.098–0.255 μg·kg^−1^	98.1–107%	≤4.7%	[42]
Chicken meat	Ciprofloxacin, enrofloxacin, sulfamethazine, oxytetracycline, tetracycline, chlortetracycline	HPLC–PDA	SPE	Acetonitrile, methanol and trichloro acetic acid	50–500 μg·kg^−1^	0.99	12.8–50.95 μg·kg^−1^	82.4–88.1%	≤11.2%	[43]
Chicken muscles, turkey	Enrofloxacin, sulfadimethoxine, sulfamerazine, sulfamoxole, sulfamethoxazole, tylosin	HPLC–DAD	SPE	Acetonitrile, methanol and acetic acid	0–1000	≥0.996	5.37–55.4 μg·kg^−1^	80.61–102.23%	≤0.98	[44]
Chicken	Clarithromycin, azithromycin, roxithromycin, kitasamycin, tylosin, tilmicosin,	LC-MS/MS	SPE	Methanol and acetonitrile	1–100 μg·kg^−1^	≥0.9942	0.2–0.5 μg·kg^−1^	82.1–101.4%	≤11.1	[45]
Duck meat	Sulfonamides, fluoroquinolones, macrolides, lincosamides trimethoprims, tetracyclines, penicillins	LC–MS/MS	SPE	Acetonitrile, methanol, trichloro acetic acid and hexane	50–500 μg·kg^−1^	≥0.9823	1.63–8.65 μg·kg^−1^	69.8–103.3%	≤6.9%	[46]
Chicken meat	Clarithromycin, erythromycin, sulfamethoxazole, sulfadimethoxine, enrofloxacin	LC-MS/MS	NADES-DLLME	Ethanol, anisaldehyde and decanoic acid	0.125–50 μg·kg^−1^	≥0.9982	0.001–0.023 μg·kg^−1^	84.1–99.4%	≤5.75	This study

**Table 5:** Molecular formulas, retention times and optimized MS/MS conditions of the target analytes.

Analyte	Retention Time (min)	Precursor Ion	Product Ion 1 (*m*/*z*)	Collision Energy (eV)	Product Ion 2 (*m*/*z*)	Collision Energy (eV)
CLR	4.32	748.3	158.2	−22	83.2	−20
ERY	4.02	734.3	158.3	−20	576.3	−20
SMX	2.54	253.9	92.1	−13	156.1	−12
SDM	3.17	310.9	156.2	−15	92.1	−12
ENR	1.82	360.0	316.2	−10	342.2	−10
OFX-d_3_	1.69	365.0	321.3	−10	261.1	−13
SMX-^13^C_6_	2.55	260.0	162.2	−13	114.3	−25

## Data Availability

The data presented in this study are contained within the article.
